# On the Properties of New Polyurethane Fast-Curing Polymer Materials

**DOI:** 10.3390/ma17246231

**Published:** 2024-12-20

**Authors:** Huachao Liu, Jiajun Deng, Shuchen Li, Richeng Liu, Liyuan Yu, Linjian Ma

**Affiliations:** 1 State Key Laboratory of Explosion & Impact and Disaster Prevention & Mitigation, Army Engineering University of PLA, Nanjing 210007, China; lhc010136@163.com; 2 School of Mechanics and Civil Engineering, China University of Mining and Technology, Xuzhou 221116, China; scli@cumt.edu.cn; 3 State Key Laboratory of Intelligent Construction and Healthy Operation and Maintenance of Deep Underground Engineering, China University of Mining and Technology, Xuzhou 221116, China; liuricheng@cumt.edu.cn (R.L.); yuliyuan@cumt.edu.cn (L.Y.)

**Keywords:** polyurethane polymer materials, temperature, particle size, proportion

## Abstract

A sequences of unconfined compressive strength tests and flexural tests were conducted in this study to evaluate the curing performance of a new type of polyurethane sand fast-curing polymer material. The mechanical properties of the material were investigated under different curing temperatures (−10 °C to 60 °C), particle sizes (10–15 mesh, 60–80 mesh, 100–120 mesh, and 325 mesh), and material proportions (20% to 60%). Additionally, SEM analysis was employed to further reveal the reinforcement mechanism. The results demonstrated that the developed polyurethane polymer material exhibited superior curing properties and applicability across a wide temperature range of −10 °C to 60 °C. Both the compressive strength and flexural strength of the solidified sand increased with the increase in solidification temperature, resulting in improved curing effects. This material exhibited the best curing properties when using sand within the 100–120 mesh range. As the particle size decreased under the remaining specifications, there was a reduction in specimen strain and an increase in strength, while still maintaining favorable ductility. The optimal proportion for polyurethane material was 40%. Moreover, the nonlinear mathematical relationships between the strength and multiple influencing factors were established through multivariate regression analysis. The sand consolidation specimens exhibited X-shaped conjugate shear failure, which tended to occur at the weak interface between the sand and material. Lastly, Pearson’s correlation analysis revealed a strong positive correlation between temperature and material content with strength.

## 1. Introduction

In recent years, China has made significant progress in vigorously promoting and executing island and mudflat projects, forming a relatively comprehensive system for protection and habitation. However, due to the influence of water flow, human activities, and other geological forces, such projects may have adverse effects or even serious consequences for long-term safety, including the leakage of sandy soil foundations and the instability of sandy slopes. Therefore, it is imperative to treat the soil according to engineering requirements, improve its physical and mechanical properties, and prevent engineering accidents. The use of sand solidification agents is a common practice in infrastructure construction [1,2,3].

For decades, cementitious materials such as lime and cement have been utilized to solidify various types of soil, providing a cost-effective solution for infrastructure construction [4,5]. Solidification can improve the strength, reduce the permeability, and even change the microstructure of sand. However, the texture of sand after solidification with cement or lime is prone to brittleness and cracking due to shrinkage or dynamic loading, which constitutes one of the primary forms of damage in rigid and flexible pavements. Meanwhile, this widespread use of materials would contribute to the greenhouse effect by releasing carbon dioxide [6,7]. Regarding this, traditional lime and cement may not always meet modern engineering requirements in certain situations, particularly for emergency repairs, even for bridge and pavement decks, airport runways, and island reclamation projects [8].

Chemical grouting materials have been widely adopted to improve the physical and engineering properties of sand due to their unique advantages, meeting diverse requirements in engineering practice [9,10,11,12,13]. Polyurethane has garnered significant attention among engineers and researchers owing to its exceptional high- and low-temperature performance, remarkable mechanical properties, outstanding permeability, simple grouting technology, high operability of raw material proportioning, and controllable curing time [14]. In addition to the commonality of chemical grouting materials, polyurethane materials also have special properties, such as rapid reaction, volume expansion, and high expansion force during polymerization. Polyurethane materials can be customized for different application scenarios and possess numerous characteristics which are highly favored in engineering applications. Polyurethane is a linear block copolymer formed by the polymerization reaction of isocyanate and polyol, with alternating arrangement of soft segments (increasing toughness) and hard segments (enhancing stiffness) [15]. After combining various material components, a spatial network structure is formed among sand particles to achieve cementation and bonding effects, thereby accomplishing the objective of improving soil strength. The polyurethane material exhibits moderate viscosity, strong corrosion resistance, and excellent adhesion performance and durability, meaning that it can be extensively applied in diverse engineering fields such as crushing structure reinforcement, dam leakage prevention, concrete repair, road maintenance, and foundation seepage prevention [16,17]. Meanwhile, polyurethane solutions can fulfill the demands for emergency treatment in terms of strength, solidification time, and waterproofing effect. Therefore, they have been adopted as materials for emergency construction rescue and long-term storage in practical applications to address sudden safety accidents [18].

In recent years, a series of tests have been conducted on the physical and mechanical properties of this type of material, which is of great significance for guiding basic engineering construction. The use of polyurethane curing agents has gradually become a hot research topic for geological engineering scholars as it can ameliorate and improve the bearing capacity, and even fundamentally change the structure of sand [19,20,21,22,23,24,25]. The polyurethane sand curing agent has extraordinary potential in enhancing the stress–strain and strength properties of sand [26,27]. Through elastic deformation, it is capable of mitigating impact, attenuating vibration, and reducing stress amplitude. This can effectively decrease the settlement of the solidified foundation under identical stress conditions [28,29]. It also has exceptional adaptability in harsh environments. The impact of polymer and sand mixing methods, polymer proportion, and curing temperature on the properties of treated sand were also taken into consideration [30,31,32,33]. The mixing order of sand and polymer has no significant effect on the strength of solidified sand. Within a certain range, the strength of the solidified sand advances approximately linearly with the increase in polymer addition, and is highly dependent on the polymer matrix [28,30]. All of these presented favorable ductility. Some scholars have pointed out that the content of polyurethane should be controlled at 15–25 wt% in asphalt [34]. Furthermore, even under extreme temperature conditions, the incorporation of polyurethane materials can improve the compressive strength and toughness of sand. Moisture content is a major inducement for the stiffness and ductility of sand consolidation under different temperatures [35]. Although polyurethane materials have been adopted in some fields, theoretical research still lags behind current engineering practices [36].

In this paper, a new type of low-viscosity, high-infiltration, fast-consolidation, high-strength, and environmentally friendly polyurethane sand-curing polymer material was designed and developed, aiming at addressing the reinforcement problem of coastal sand foundation. The mechanical properties and sand consolidation properties of the polymer material were investigated by altering the curing temperature, sand particle size, and material proportion. This breakthrough technology overcomes the limitations of conventional foundation reinforcement materials, such as poor infiltration, slow molding speed, insufficient mechanical properties, and complex processes. In addition, it provides essential data and theoretical support for rapid reinforcement, emergency repair and construction, and reclamation.

## 2. Experimental Materials and Procedure

### 2.1. Sand

**Figure 1:**
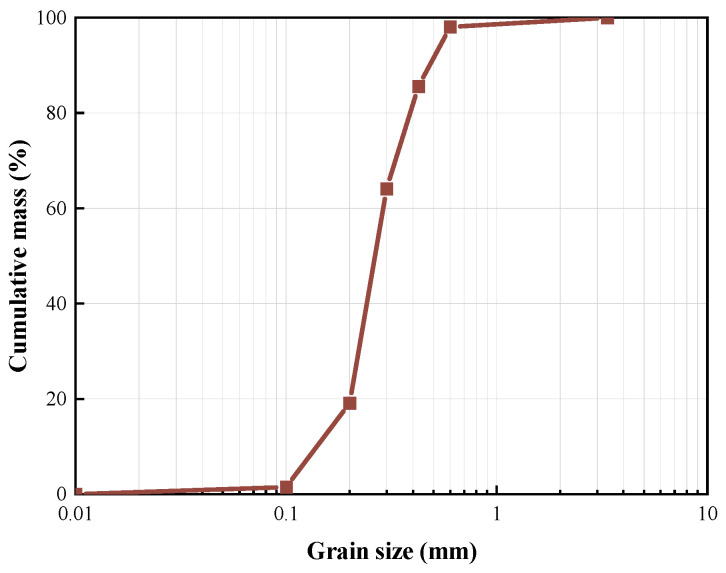
Distribution of sand grain size.

**Figure 2:**
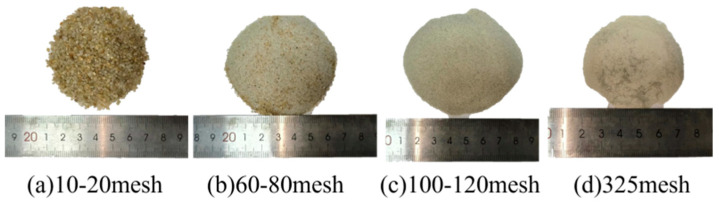
Sand with different grain sizes.

The grading curve of Fujian sand used in those experiments is illustrated in Figure 1. It has been established that the porosity of the sand samples collected on-site is approximately 22%, with a moisture content of around 24%. The primary constituent of the natural sand utilized in the experiment is silicon dioxide, which comprises approximately 92%. To investigate the impact of different sand grain sizes on material curing performance, the sand was washed with running water and dried at a temperature of 105 °C for eight hours. The dried sand was left to stand for one day before screening. The resulting sand with varying grain sizes obtained after screening is shown in Figure 2.

### 2.2. Polyurethane Polymer Materials

**Figure 3:**
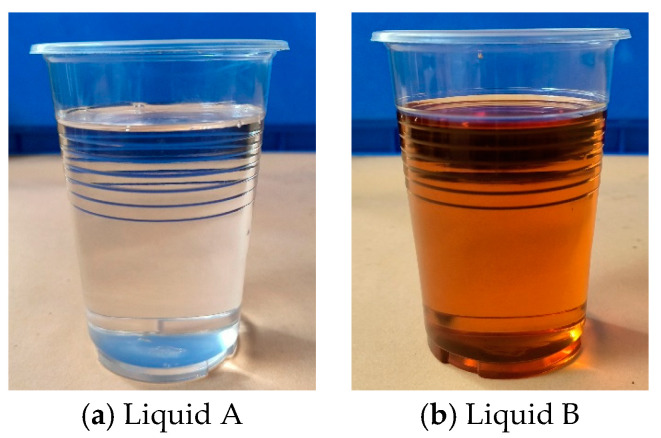
Non-isocyanate polyurethane.

The new sand fast-curing agent used in this article is a non-isocyanate polyurethane material, which has the advantages of high strength, low viscosity, and fast solidification speed. The material was synthesized by combining two component solutions, A and B, at a volume ratio of 1:1 through ammonolysis and polymerization addition reaction (Figure 3). Traditional polyurethane materials contain toxic polyisocyanate, which is not conducive to the construction of public facilities. By contrast, the polyurethane material investigated and utilized in this article not only circumvented the use of toxic reagents but also exhibited eco-friendly characteristics.

Solution A was a colorless and transparent liquid obtained by mixing cyclic carbonate with epoxy resin in a 4:1 ratio. The synthesized cyclic carbonate had the advantage of low viscosity, which can effectively penetrate and reinforce the sandy soil foundations through dilution with the polyurethane material. Additionally, it promoted the formation of cross-linked network structures in non-isocyanate polyurethane materials and further enhanced the mechanical properties of the materials. Solution B was a brown solution, which was composed of three types of polyamines, Diethylenetriamine, Aromatic amine, and long-chain aliphatic amine, constituting the compounded polyamine curing agent as the main agent of solution B. These amine compounds possess diverse structures and functions, providing strong controllability over material properties. Among them, Ethylenediamine and Diethylenetriamine are effective in reducing material viscosity and promoting material penetration due to their low molecular weight and viscosity. In addition, they can enhance the cross-linking density of materials, improving the mechanical properties of polyurethane materials and their sand consolidation. Aromatic amine exhibited high reactivity and contained a rigid benzene ring, which can be incorporated into the product structure post-reaction to improve the rigidity of the material. Long-chain aliphatic amines can improve the toughness of materials [37].

### 2.3. Experimental Procedure

#### 2.3.1. Specimen Fabrication

**Figure 4:**
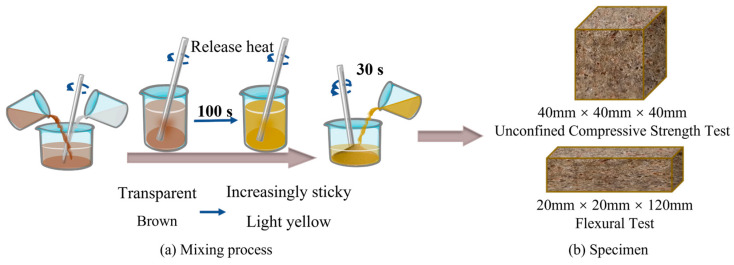
Specimen fabrication process.

Following the established quality, the A and B component materials were mixed at a volume ratio of 1:1, using a glass rod for 100 s at a temperature of 25 °C. As an obvious exothermic reaction occurred, the solution changed from transparent light brown to viscous and light, with small bubbles forming in the liquid. The mixture was then poured into pre-weighed sand and fully mixed in a mixing container for 30 s before being poured into the mold (Figure 4) (without adding sand when studying the mechanical properties of raw material strength). The inner surface of the mold was pre-coated with Vaseline and attached with 0.1 mm PTFE film to isolate the specimen. The bottom of the mold was installed with a PTFE pad. After the sand was poured into the mold, it was speedily vibrated 3–5 times with a vibrator rod. The mixed sand was added during each vibration to fill up the mold. After the process of vibration and compaction, a geotechnical knife was promptly utilized to flatten any protruding parts along the surface of the mold. The mold was placed in a fixture to prevent expansion, cured for 40 min, and subsequently removed. Then, the sand consolidation specimens were subjected to mechanical performance tests.

#### 2.3.2. Test Procedure

**Figure 5:**
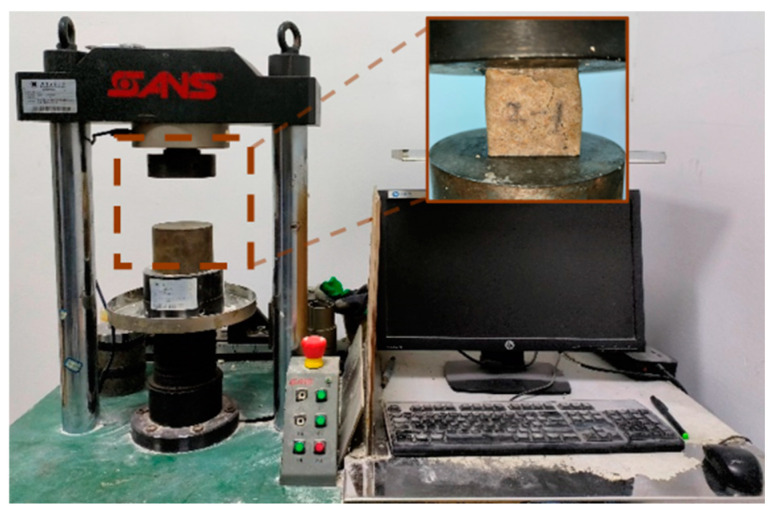
Test machine.

The experimental variables were set as follows: grain size varied between 10–15 mesh, 60–80 mesh, 100–120 mesh, and 325 mesh; material proportions, defined as the percentage by weight of the new polymer material relative to dry sand, were mixed at levels of 20%, 30%, 40%, 45%, 50% and 60%; and the temperature values were −10 °C, 0 °C, 25 °C, 40 °C, and 60 °C. The detailed test scheme is summarized in Table 1. Subsequently, the strength of the manufactured specimen was tested using an electronic universal testing machine (C51.105 of New Sansi (Shanghai) Enterprise Development Co., Ltd., Shanghai, China), as shown in Figure 5. The mechanical properties of the material were in compliance with the standard GB/T17671-1999 *Test Method for Strength of Cement Mortar* [38]. Unconfined compressive strength test specimens measured 40 mm × 40 mm × 40 mm, with a loading rate of 0.24 mm/min. Flexural test specimens were 20 mm × 20 mm × 120 mm, with the compression surface as the side located at a distance of 100 mm from the fulcrum, and the loading at 0.24 mm/min. Testing was performed until the specimen was destroyed or the pressure dropped below 70% of the maximum stress value, and the maximum stress value was recorded. Three parallel samples were prepared for each proportioned specimen, and the mean value was subjected to analysis. In addition, the correlation analysis and statistical assessment were utilized to analyze the relationship between material parameters (grain size, material proportions, and curing temperature) and strength properties [39,40,41].

## 3. Results and Analysis

### 3.1. Raw Material

**Figure 6:**
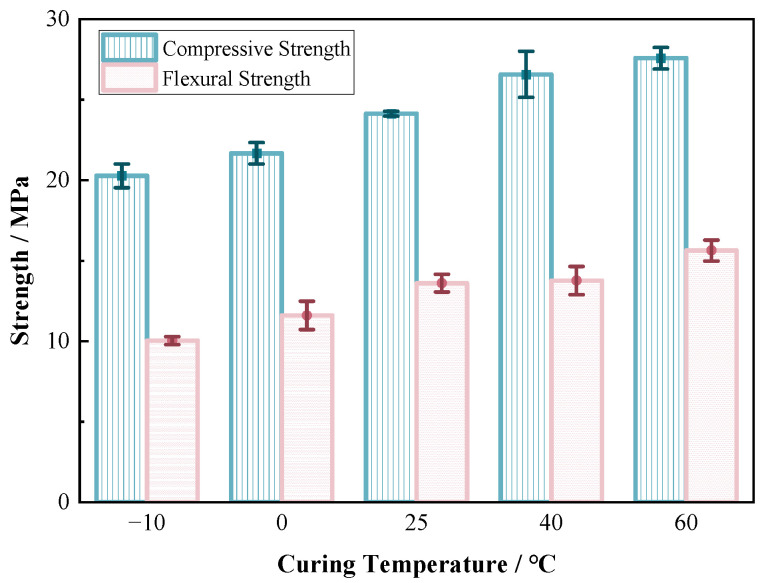
Compressive strength/flexural strength of raw materials at different temperatures.

According to Figure 6, the compressive strength of the raw material, solidified for 40 min at environmental temperatures of 60 °C, 40 °C, 25 °C, and 0 °C, was measured as 28 MPa, 26 MPa, 24 MPa, and 22 MPa, respectively, while the flexural strength was recorded as 15 MPa, 14 MPa, 13 MPa, and 12 MPa, respectively. Even when the temperature dropped down to −10 °C, after curing for 40 min, the raw material still exhibited compressive strength and flexural strength of up to 20 MPa and 10 MPa. This indicated that the material had a fast and effective curing effect, while maintaining high strength across various temperature ranges. Within the specified temperature range, every 1 °C increase in temperature resulted in an incremental compressive strength of 0.08 MPa to 0.2 MPa for the raw material specimen. Although the increase in strength was not significant, higher temperatures led to a more complete reaction between components A and B of the solidification agent, resulting in an enhancement in both compressive strength and flexural strength. However, the impact on compressive strength was relatively more pronounced.

### 3.2. Sand Consolidation

The specimens were subjected to an unconfined compression test and flexural test, using six kinds of material proportions, four grain sizes, and five curing temperatures. The detailed analysis of each part is as follows.

#### 3.2.1. Curing Temperature

Temperature was a critical factor that significantly affected the curing and mechanical properties of materials. Therefore, it was particularly pertinent to investigate the impact of curing temperature on the mechanical properties of sand consolidation.

**Figure 7:**
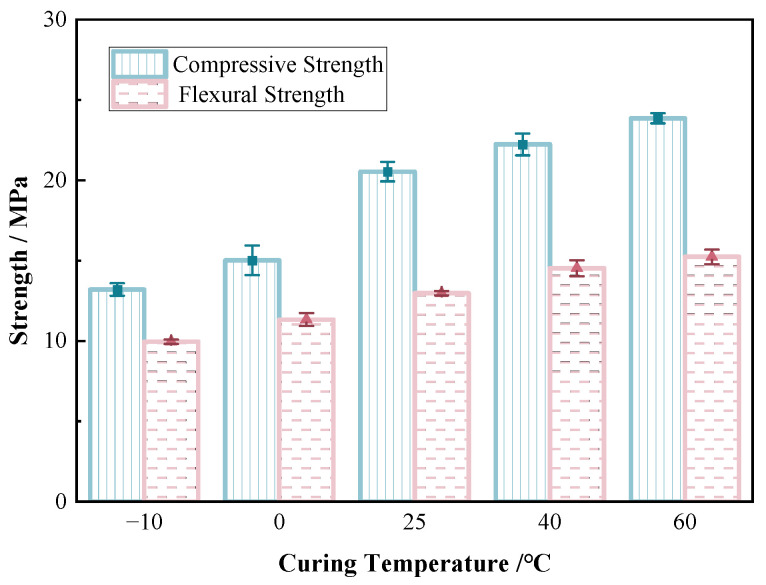
Compressive strength/flexural strength of sand consolidation at different temperatures.

As depicted in Figure 7, the consolidation strength of sand (the material content was 20 wt% of the sand) exhibited a similar trend to that of the raw material at different temperatures. After 40 min of curing, the compressive strength of the sand consolidation was measured at an ambient temperature of 60 °C, 40 °C, 25 °C, 0 °C, and −10 °C, resulting in values of 24 MPa, 22 MPa, 20 MPa, 15 MPa, and 13 MPa, respectively. At the same time, the flexural strength was also measured at 15 MPa, 14 MPa, 13 MPa, 11 MPa, and 10 MPa, respectively. Among them, unlike the raw material, the temperature increase from 0 °C to 25 °C resulted in a more notably increase in the strength of sand consolidation specimen, with growth rates of compressive strength and flexural strength at 33.3% and 18.2%, respectively. As temperature increased, the solidification of sand gradually advanced, leading to the improved strength and stiffness of the material. The influence of temperature on the strength performance of the specimen was primarily observed in the presence of residual water during the chemical reaction process of the material [42], which influenced both the strength and ductility of the polymer film (a spatial network structure formed between the sand particles after each component reaction of the polymer material), ultimately affecting the stiffness and integrity of sand consolidation. The properties of the polymer curing film were evidently affected by temperature, as evidenced by thermal expansion behavior. At lower temperatures, the connection between the curing film and sand particles would relax or even peel off due to shrinkage, resulting in a decrease in the strength of the solidified sand. Additionally, the limited evaporation of residual water occurred within samples at lower temperatures. However, under high environmental temperatures, the water content within the sample could further evaporate, causing a decrease in water content within the polymer curing film. Therefore, this enhanced the strength of the polymer curing film and led to an increase in the strength of the solidified sand sample with increasing temperature. Nevertheless, this increase in strength was also restricted with excessive temperature, which can accelerate the fracture and detachment of the film.

However, the mechanical properties of sand consolidation were slightly inferior to raw material due to the fact that the mechanical properties of sand consolidation mainly depended on polymer materials. The fluidity of reactants was a crucial factor in the reaction of polymer materials, which was similar to the solidification process of concrete in water. Temperature affected the liquid composition and thus altered the properties of sand. When the organic phase polymer material was mixed with sand, the inherent properties of the two phases were really different. The reduction in organic component content resulted in a weaker consolidation strength of sand than that of raw materials. Simultaneously, a large number of tiny bubbles formed on the surface of the sand during polymer reaction, which were difficult to discharge. The presence of bubbles in the cured polyurethane solution resulted in stress concentration, which reduced the compressive strength and flexural strength of the sand consolidation to a certain extent. However, the material had favorable permeability and the ability to fully fill gaps between the sand grains, and there was no marked decrease in mechanical properties.

#### 3.2.2. Grain Size

The new polymer material had excellent penetration performance across various grain size specifications. An investigation was conducted into the relationship between the sand consolidation properties and the sand fineness, with 20 wt% content of the material, and the curing temperature at room temperature (25 °C).

**Figure 8:**
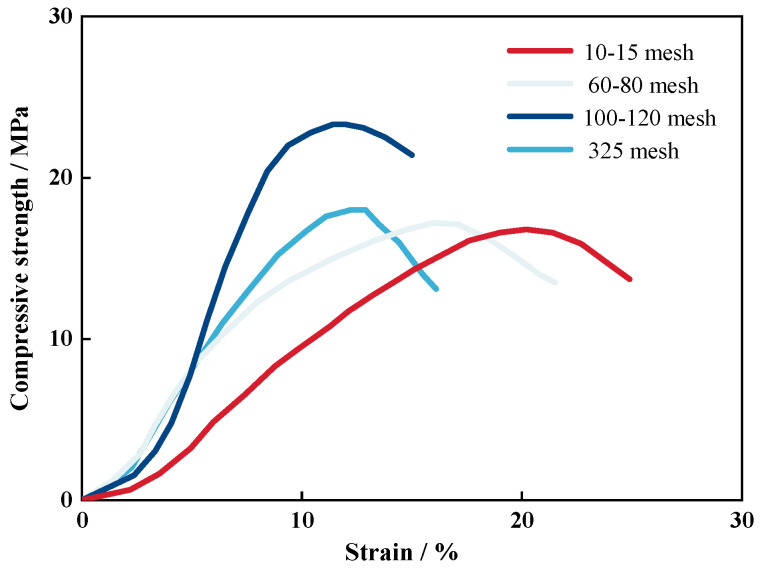
Compressive stress–strain curves of sand consolidation with different particle sizes.

From Figure 8, it was evident that the stress–strain behavior of the sand consolidation gradually transitioned from stress hardening to strain softening. It was noteworthy that the stress–strain response of sand consolidation was different from that of the cement treatment, as it exhibited greater ductility and did not suddenly drop in strength. This property has significant implications for controlling foundation settlements in engineering applications [43,44]. With an increase in sand particle size, there was a gradual rise in the deformation of the sand consolidation. This was attributed to smaller sand particles being more prone to accumulate and compact, thereby more closely approaching the continuous phase. With the exception of the particle size range of 100–120 mesh, which was higher than 0.365 GPa compared to other particle sizes, the elastic modulus gradually increased as particle size decreased in the remaining three ranges (Table 1). Macroscopically, this trend indicated an increasing rigidity in sand consolidation. The curing network formed by the reaction between each component of the material provided a bridge connection across the gaps between sand particles, exhibiting strong adhesion and preventing the brittle failure of the sand consolidation specimen. On the other hand, polymer materials could easily fill the pores or completely wet the sand within the optimal particle size range of 100–120 mesh due to the large gap between sand particles. A relatively dense solidification film could be formed between the material and sand, resulting in improved rigidity compared to sand consolidations produced under other specifications. Therefore, this sand consolidation specimen demonstrated superior mechanical strength [43,44].

**Figure 9:**
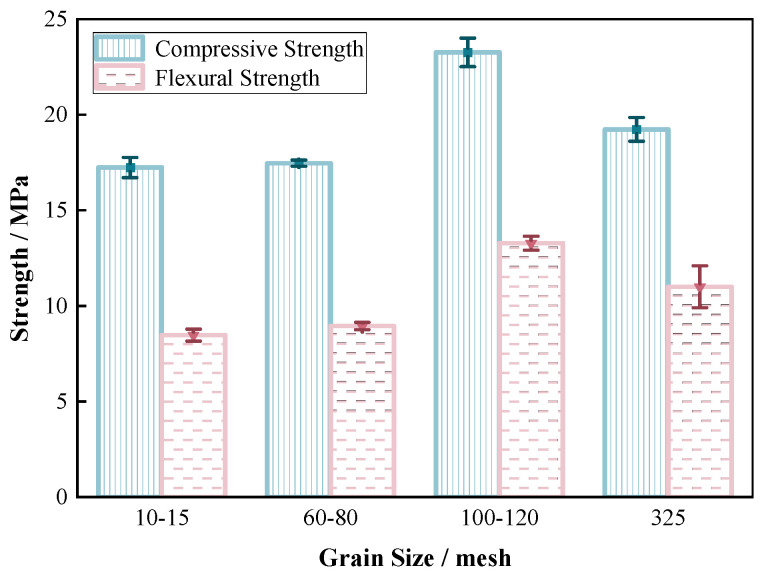
Compressive strength/flexural strength of sand consolidation with different particle sizes.

Based on Figure 9 and Table 2, it was revealed that the compressive strength and flexural strength of the new fast-curing polymer material sand consolidation exhibited an increasing trend with an increase in mesh number under the conditions of 10–15 mesh, 60–80 mesh, and 325 mesh. Although a single gradation might slightly weaken the strength of sand consolidation, compared with conventional cementitious materials, the sand fast-curing polymer material exhibited minimal selectivity towards sand. The strength of the consolidated specimen was primarily provided by the polymer material, resulting in a significant decrease in strength. When the sand fineness was under the condition of 100–120 mesh, it displayed the most superior solidification effect with a compressive strength of 23.3 MPa and a flexural strength of 13.3 MPa. The test results for sand consolidation within this particle size range surpassed those of other mesh sizes due to its moderate specific surface area and porosity. The sand particles in this range had moderate properties, and polymer materials demonstrated better filling and bonding effects. Moreover, the excellent crystalline state of quartz sand enhanced the mechanical strength of the sand consolidation. In this size range, the effective contact area between the material and the sand particles was relatively large, which directly impacted the interfacial friction and adhesion. Therefore, this sand consolidation specimen exhibited the highest mechanical strength [43,44]. The sand consolidation specimen prepared with larger particle size sand contained more voids between particles, and had a majority of point contacts and weak points under stress, which did not facilitate significant improvement in strength. Additionally, excessively fine particle size sand possessed a large specific surface area that made it difficult for the solution to completely wet all the particles. Therefore, the strength of the sand consolidation specimen prepared by it was revealed to be lower than that of 100–120 mesh sand consolidation specimen.

#### 3.2.3. Material Proportions

The mechanical properties of sand consolidation were primarily provided by polymer materials, thus making material proportion a critical factor in determining the mechanical properties of sand consolidation. The sand consolidation specimens prepared in this section were all cured by mixing 60–80 mesh sand with the material.

**Figure 10:**
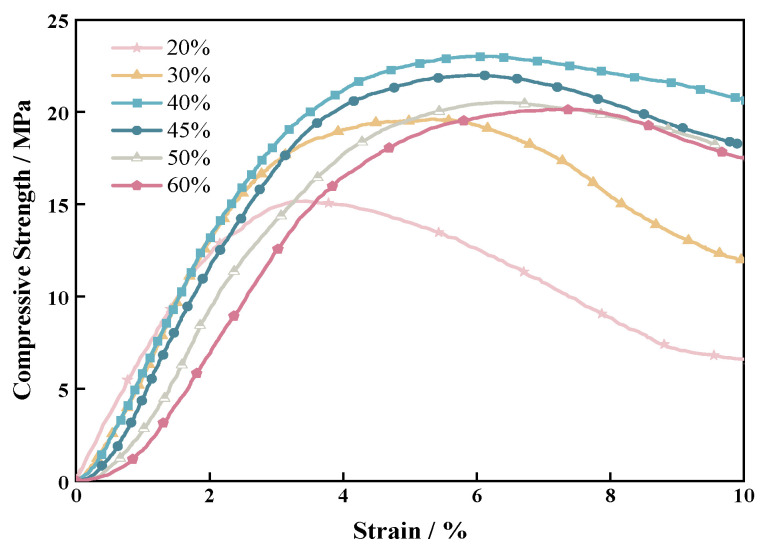
Compressive stress–strain curves of sand consolidation with different material proportions.

**Figure 11:**
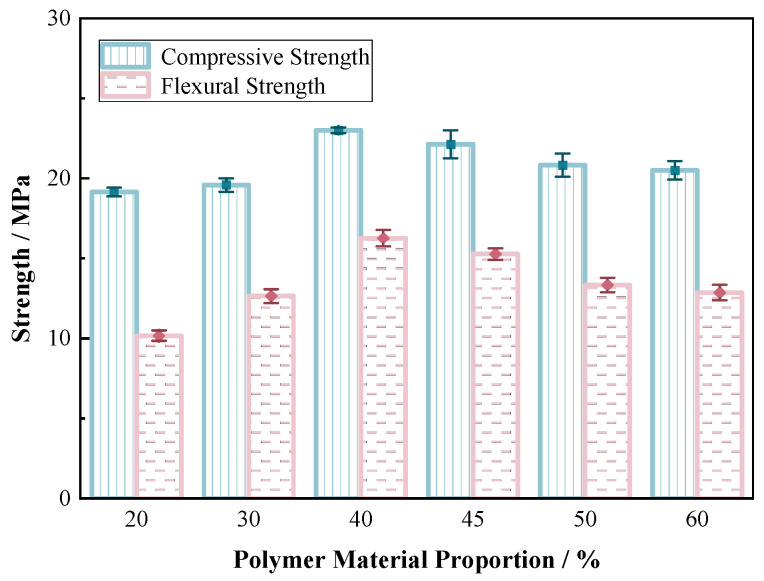
Compressive strength/flexural strength of sand consolidation with different material proportions.

Figure 10 and Figure 11 demonstrate that all specimens in this experiment exhibited strain-softening plastic failure, which refers to a phenomenon where the material’s strength decreases with increasing strain after reaching peak stress, ultimately leading to gradual failure. Initially, the curves of samples with different polymer contents almost overlapped, indicating an obvious compaction stage, which may be attributed to the incomplete operation of the polymer film at the beginning. The axial stress reached its maximum as the axial strain increased, and subsequently decreased gradually to a relatively stable value with further increasing strain. As the strain increased, the stress–strain curve of the high polymer content specimens became wider and flatter, indicating enhanced ductility. The higher the content of the polymer curing agent, the thicker the resulting film formed on sand, with higher elasticity and ductility. This material exhibited significant improvements in strength and toughness as temperature increased, ultimately causing a hardening phenomenon in the improved sand. At the same dry sand density, an increase in polymer concentration led to a higher axial failure strain of the reinforced specimen, exhibiting better ductility. Even with a large amount of polymer material, it could still maintain a significant strain, demonstrating the excellent flexibility of the material.

With an increase in material proportion, the compressive strength and flexural strength of the sand consolidation layer firstly increased. At the proportion of 40%, the strength decreased with further increase in the proportion until it eventually plateaued. This experiment demonstrated that 40% was the optimal proportion for the new type of sand fast-curing polymer material, resulting in maximum strength. When the material content was below 40%, increasing the proportion further filled the gaps between sand particles, forming a dense particle arrangement that gradually solidified the sand structure and provided stronger constraints. By enhancing the structural bonding and aggregation among sand particles, a high-strength and -toughness curing network was formed. As the content of polymer curing agent increased, the ability to resist deformation was strengthened and the rigidity increased. The maximum increase in strength occurred when the material content increased from 30% to 40%, with compressive strength and flexural strength growing by 17.9% and 28%, respectively. Simply increasing the polymer content did not necessarily enhance the strength of the specimen. This was because excessive polymer would accumulate on the outer surface of the specimen due to limited space at a given dry density, which hindered the improvement in sand structure [45]. Therefore, an optimal proportion for polymer material must be maintained to prevent the uneven suspension or precipitation of sand particles in the polyurethane solution, which can lead to stress concentration points and a decrease in the strength of the sand consolidation when the material content exceeds 40%. The strength characteristics of improved sand were significantly influenced by the content and temperature of polymer curing, and compressive strength was more sensitive to temperature when a higher amount of polymer curing agent was present. Compared to the effect of temperature on sand consolidation, the addition of polymer materials for fast solidification had a greater impact on flexural strength.

#### 3.2.4. Prediction Model of Estimation of Strength

Based on the aforementioned experimental data, it can be inferred that the compressive strength and flexural strength of sand consolidation are influenced by parameters such as curing temperature, sand grain size, and material proportion [46,47]. Therefore, the paper conducted multivariate regression analysis separately for compressive strength and flexural strength to establish two independent nonlinear mathematical relationship models:

(1) $$\begin{array}{l} CS=34.9+0.19TEM-24.22GS-52.14MP+\\ \;9.33GS^{2}+200.87MP^{2}-1.02GS^{3}-205.58MP^{3}\;\\ R^{2}=0.93 \end{array}$$

(2) $$\begin{array}{l} FS=22.90+0.03TEM-22.67GS-5.05MP+\\ \;8.22GS^{2}+66.68MP^{2}-0.87GS^{3}-109.66MP^{3}\;\\ R^{2}=0.84 \end{array}$$

where *CS* and *ZS* represent compressive strength and flexural strength; and *TEM*, *GS*, and *MP* denote the curing temperature, grain size, and material proportion, respectively. And the values of the coefficient of determination *R*^2^ are 0.93 and 0.84 for *CS* and *ZS*, respectively. Hence, the mathematical model could be utilized to predict the consolidation strength of sand under the aforementioned conditions.

### 3.3. Failure Mode and Failure Mechanisms

#### 3.3.1. Failure Mode

**Figure 12:**
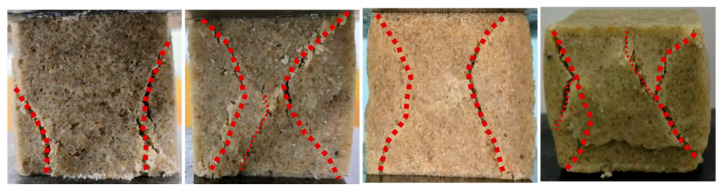
Failure mode of specimens with different material proportions. (The red lines indicate the development of cracks).

Figure 12 illustrates the failure mode of standard sand specimens under uniaxial compression with varying material proportions, all exhibiting a typical X-shaped conjugate shear failure pattern characterized by intersecting shear planes forming an “X” shape. The polymer matrix among sand particles was subjected to compressive, tensile, shear, and other forces, all of which were destructive to the structure [42,48]. In the initial stage of increasing compressive load, the sand consolidation specimens were in a compacted state. The polyurethane matrix formed an elastic connection among sand particles, distributing stress to a larger area through effective contact and preventing damaged due to high local stress. Therefore, only the height of the specimens changed. With an increase in material proportion, the height of the specimens which compressed within the same time period exhibited more pronounced change, thus confirming the above-mentioned rule that the higher polyurethane content led to better ductility in sand consolidation. As the growth of compressive load, friction occurred at both ends of the specimen and lateral deformation was limited by the Poisson effect. Only the central part of the specimen experienced lateral deformation, forming two symmetrical compression regions on the front and rear surfaces of the specimen, which led to the development of microcracks inside the specimen. The compressive stress continuously increased and extended towards the interior of the specimen, gradually evolving into an X-shaped conjugate shear failure crack with a certain apparent length and width until the crack propagated and penetrated. The surface of the specimen in the shear failure area experienced detachment, ultimately resulting in damage to the specimen.

#### 3.3.2. Failure Mechanism

**Figure 13:**
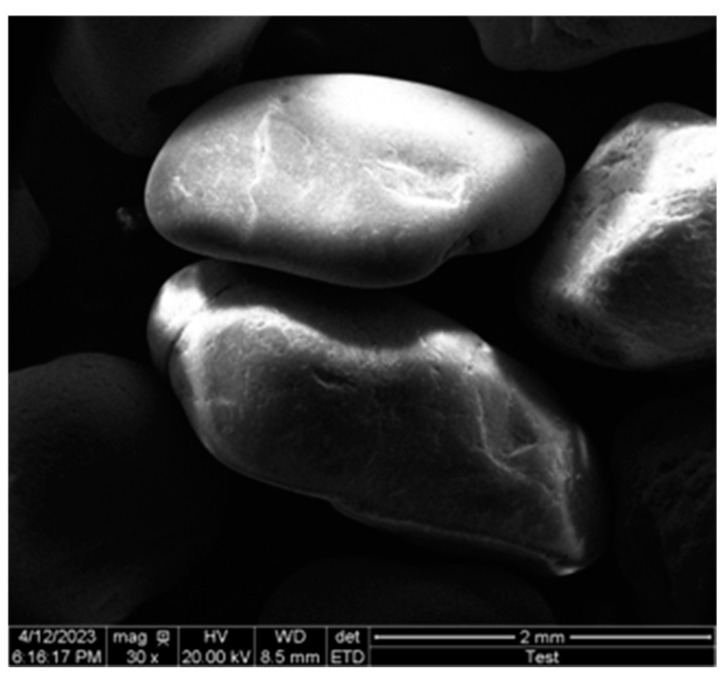
SEM images of particle.

**Figure 14:**
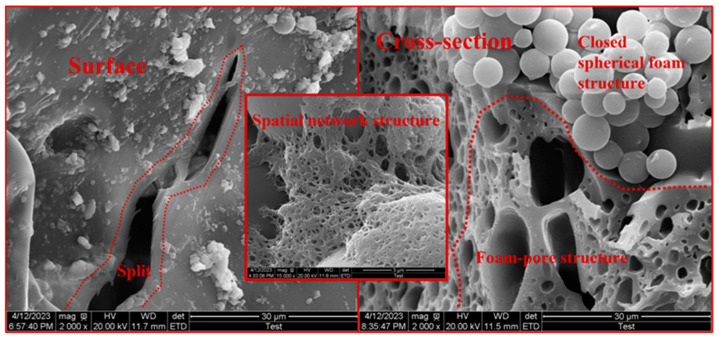
SEM images of function mechanism of polyurethane polymer material.

The microstructure of the particles can directly or indirectly affect the adhesion of polymers to sand particles. The surface of sand particles was relatively smooth (Figure 13), mostly in point contact and rare in surface contact, resulting in poor interlocking and adhesion effects between unconsolidated sand particles, which was prone to sliding and particle rearrangement. The curing process of polyurethane materials cemented sand particles closer, causing an increase in the occlusal force between particles and resistance to rearrangement. The primary mechanism of action for polyurethane materials was as follows (Figure 14). The material underwent an exothermic reaction upon exposure to air, resulting in the formation of a spatial network structure that cemented loose sand particles together. Meanwhile, the foaming agent molecules experienced a phase change from liquid to gas, generating closed spherical foam structures that filled the interstitial spaces between and within individual particles. The structure generated by the reaction enhanced specimen cohesion and integrity, while also filling the gaps between sand particles to improve compactness and limit lateral deformation. It played a crucial role in resisting external forces and providing channels for external stress. During the initial compaction stage, the foam–pore structure formed by material reaction was compressed. The unique structure produced by the reaction of polyurethane components exhibited excellent tensile and diffusion effects, imparting superior elasticity and flexibility to sand consolidation. Consequently, this structure could undergo significant deformation without fracturing under external forces, thereby enhancing its mechanical properties. As the temperature increased, the cured film produced higher strength and ductility through shrinkage, resulting in an increase in both compressive and flexural strength. Moreover, excessively high temperatures can accelerate film rupture and the detachment of sand particles [45].

**Figure 15:**
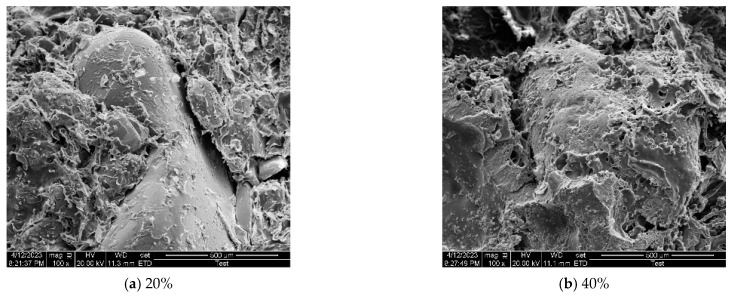
SEM images of specimens with different material proportions.

Figure 15 revealed that the binding and cementation effect on sand particles improved with increasing polyurethane content when the material content was less than 40%. The addition of polymer material to sand caused matrix reactions that adsorbed on the surface of the sand particles through hydrogen bonds or intermolecular forces, which was macroscopically manifested as the formation and attachment of a curing film on the particles. This film tightly combined smooth sand particles, providing the specimen with higher ability to resist external forces and further improving the strength of the sand consolidation specimens. Moreover, the pores among the particles were filled with a thin film that became more complete as the material content increased, ensuring the overall formation and stability of the specimens. The higher the density of the curing agent matrix within the sample, the greater the contact area between the polymer and the sand particles. This resulted in a denser curing film and improved the cement effect, leading to the enhanced cohesion of sand consolidation and superior curing effects [28]. Therefore, the mechanical properties of the sand were substantially enhanced. This film was a lightweight and flexible material with remarkable buffering capabilities and effective energy transfer mechanisms. By absorbing and mitigating impact through elastic deformation, it enabled an appropriate stress distribution by effectively transmitting stress through sand particles. However, excessive material content can lead to the random suspension and uneven distribution of sand particles within the material, creating stress concentration points that might affect the intended benefits of the curing film.

The solidified specimens with varying proportions initially detached at the cemented interface (Figure 15). As the axial force increased, the material displayed different degrees of fracturing, which exposed internal pore structures. The reinforcement effect of the polymer material on the specimen was primarily determined by the interlocking force between sand particles and materials, as well as the cemented strength at the interface. Therefore, enhancing the cemented state of interfaces played a crucial role in improving sand consolidation strength. The elastic behavior of the specimen under uniaxial compression load was primarily determined by the strength of the foam–pore structure. Loading caused foam to compress and release gas, resulting in the bending and deformation of the foam wall. Eventually, the foam wall experienced stretching and cracking (Figure 14). The smaller position of spherical foam was susceptible to stress concentration and strain localization. The non-uniform foam–pore distribution caused heterogeneous stiffness. When subjected to compressive loading, some weak foam–pore structures experienced significant deformation, ultimately leading to the macroscopic failure of the specimen.

## 4. Discussion

To investigate the correlation between various influencing factors and compressive strength, the *Pearson correlation coefficient* (*R*) was utilized as an evaluation indicator to analyze the relationship between its strength and the relative increment of sand particle size, environmental temperature, and material content. A correlation coefficient matrix was obtained. In the equation below, *Cov*(*X*, *Y*) represents the covariance between *X* and *Y*, while *Var*[*X*] and *Var*[*Y*] denote the variances of *X* and *Y*, respectively. The correlation coefficient *R* was in the range [−1, 1]. The higher absolute value indicating a stronger correlation between *X* and *Y*. Conversely, a lower absolute value suggests a weaker correlation between the two variables.

(3) $$R=\frac{Cov\left(X,Y\right)}{\sqrt{Var\left[X\right]Var\left[Y\right]}}$$

**Figure 16:**
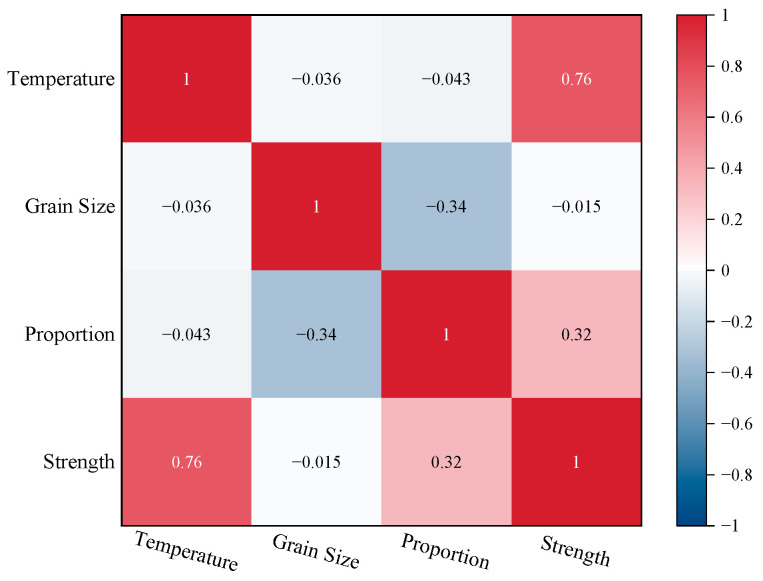
Correlation matrix of each specimen factor.

The correlation coefficient matrix is depicted in Figure 16. It was evident that there existed a strong positive correlation between temperature and material content with strength, while the impact of strength was more sensitive to changes in temperature. The increase in temperature was accompanied by a corresponding increase in the strength of the sand consolidation specimens, demonstrating the material’s suitability for using in various environments, even in extreme areas. Although there existed an optimal material proportion within the range of polyurethane curing agent content that was considered comprehensively in practical engineering, a higher material proportion produced better curing effects. There was a negative correlation between sand particle size and compressive strength, although the effect was not significant. Based on the above analysis, an optimal particle size range of 100–120 mesh was identified. It is worth noting that there was a certain coupling effect between the material proportion and the sand particle size, with a significant negative correlation observed between these two factors. With the increasingly widespread application of polyurethane materials as sand solidification agents in practical engineering, it is worthwhile to conduct further research and exploration into the various factors which influence their mechanical properties.

## 5. Conclusions

The following conclusions were derived from using a new polyurethane sand fast-curing polymer material through unconfined compressive tests and flexural tests:

(1) The material exhibited fast curing and outstanding adaptability across a range of temperatures. Under different temperatures, the compressive strength and flexural strength of the material and the sand consolidation specimens all followed similar trends. With the increase in temperature, the sand consolidation was gradually improved. Both the compressive strength and flexural strength were enhanced with increasing temperature. Additionally, the former was more significantly affected. The strength of the material was greater than that of the sand consolidation specimens, primarily due to the presence of solidified materials.
(2) With the exception of 100–120 mesh, an increasing in particle size resulted in a gradually reduction in sand consolidation strain while maintaining excellent ductility. Both strength and stiffness increased. The 100–120 mesh range was optimal for sand grade with the best curing effect.
(3) The optimal proportion of the sand polymer material for fast curing was 40%. Initially, the compressive strength of the specimen increased and then decreased with a turning point at a 40% material content. However, the ductility of the sand consolidation specimen improved with an increase in material content.
(4) Under different material proportions of the specimen, sand consolidation displayed an X-shaped conjugate compression shear failure mode. The spatial network structure and foam–pore structure generated by the reaction of various components in polyurethane polymer materials had outstanding potential for enhancing the mechanical properties of loose sand.
(5) Two nonlinear mathematical relationships were established through multivariate regression analysis to estimate the strength. Moreover, Pearson’s correlation analysis revealed a strong positive correlation between temperature and material content with strength.

## Figures and Tables

**Table 1:** Test scheme of influencing factors.

Influencing Factors	Variation Range	Constraining Factors
Curing Temperature	−10 °C, 0 °C, 25 °C, 40 °C, and 60 °C	60–80 mesh, material proportions 20%, curing 40 min
Grain size	10–15 mesh, 60–80 mesh, 100–120 mesh, and 325 mesh	25 °C, material proportions 20%, curing 40 min
Material proportions	20%, 30%, 40%, 45%, 50%, and 60%	25 °C, 60–80 mesh, curing 40 min

**Table 2:** Experimental results of sand consolidation under different sand particle sizes.

Particle Size	Compressive Strength/MPa	Elastic Modulus/GPa	Flexural Strength/MPa
10–15 mesh	17.2	0.105	8.5
60–80 mesh	17.5	0.178	8.9
100–120 mesh	23.3	0.365	13.3
325 mesh	19.2	0.187	11.0

## Data Availability

The original contributions presented in this study are included in the article. Further inquiries can be directed to the corresponding author.
